# Association of High BMI with Dental History, Sociodemographic Characteristics, and DMFT Index in Female Students at Taif University Sports Center: A Cross-Sectional Analysis

**DOI:** 10.3390/jcm14103464

**Published:** 2025-05-15

**Authors:** Ali Abdullah Alqarni, Abeer Ali Qahtani, Amal Mohammad Albalooshy, Bandar Saud Shukr, Shaimaa Mohammed Alarabi, Fahad Saeed Algahtani, Azzah Owayimer Alhazmi, Mohammed Fareed Felemban, Amal Adnan Ashour

**Affiliations:** 1 Department of Oral & Maxillofacial Surgery and Diagnostic Sciences, Faculty of Dentistry, Taif University, Taif 21944, Saudi Arabia; aqarni@tu.edu.sa (A.A.A.); smharthi@tu.edu.sa (S.M.A.); m.felemban@tu.edu.sa (M.F.F.); 2 Department of Restorative Dental Science, Faculty of Dentistry, Taif University, P.O. Box 11099, Taif 21944, Saudi Arabia; abeer.q@tu.edu.sa (A.A.Q.); f.algahtani@tu.edu.sa (F.S.A.); 3 Department of Preventive Dentistry, Faculty of Dentistry, Taif University, P.O. Box 11099, Taif 21944, Saudi Arabia; amal.m@tu.edu.sa (A.M.A.); b.shukr@tu.edu.sa (B.S.S.); az.alhazmi@tu.edu.sa (A.O.A.)

**Keywords:** body mass index (BMI), female, habits, obesity, oral health, sociodemographic

## Abstract

**Background/Objectives:** Oral health has a significant impact on our overall well-being. The DMFT index assesses dental caries prevalence, whilst the body mass index (BMI) estimates body fat, with obesity defined as BMI ≥ 30 kg/m^2^. Obesity adversely affects women’s health, including increased risks of chronic diseases. In Saudi Arabia, with a rising obesity rate, especially amongst women, highlights the need to investigate the relationship between BMI and oral health. Our aim is to evaluate the association of high BMI (body mass index), dental history, diet, physical activity, and oral hygiene practices with DMFT (decayed, missing, and filled teeth) of female students at Taif University, Saudi Arabia. **Methods:** This cross-sectional observational study included female students at a sports facility in Taif University, Saudi Arabia, with a high BMI. A convenience sampling technique was used. Participants were categorized into three groups based on their BMI. Data was then collected through structured interviews and oral examinations. The prevalence and types of chief complaints, sociodemographic status, and DMFT index in female students with a high BMI, as well as the possible mechanisms linking BMI, were analyzed. **Results:** The study included 138 female students, 86 of whom were obese female students, aged 18–27. Compared to the control group, participants with higher BMI were more likely to visit the dentist due to pain and had a higher number of missing teeth. Systemic diseases, such as asthma and type 2 diabetes, were significantly more prevalent among obese participants. No significant correlation was found between BMI and dental hygiene practices or dietary habits. Higher BMI was significantly associated with an increase in missing teeth (β = 0.09, 95% CI: 0.00 to 0.18, *p* = 0.045). However, it was not found with the overall DMFT index. **Conclusions:** higher BMI among female university students was associated with an increased prevalence of missing teeth and systemic diseases

## 1. Introduction

The decayed, missing, and filled teeth (DMFT) index is a widely recognized epidemiological tool used to assess dental caries prevalence and oral health status in populations [1,2,3].

Body Mass Index (BMI) is used to estimate body fat based on an individual’s height and weight, with obesity defined as a BMI of 30 kg/m^2^ or higher [4]. BMI is widely recognized as a measure of overall health and risk of chronic diseases [5,6]. Obesity affects women’s health adversely [7], including general and reproductive health, such as an increased risk of diabetes, coronary artery disease, musculoskeletal issues, obstetrical conditions, and neonatal mortality and malformations, in addition to psychological aspects, such as depression, weight stigma, suicidal thoughts, and emotional dysregulation [8].

In Saudi Arabia, the prevalence of obesity has been rising, particularly amongst women, with the country ranking 29th globally, and third amongst the Arab nations, in terms of obesity prevalence [9]. Furthermore, approximately 36% of university students in Saudi Arabia have high BMI [10]. This high prevalence emphasizes the importance of studying obesity-related health issues in this population. Previous studies focused mainly on the general population or specific clinical samples and used different measures to assess oral health [1,2,9].

Chief complaints (CC) in dental settings often reflect underlying oral health issues and can serve as indicators of broader health problems [11]. Common dental histories such as toothache, sensitivity, and gum bleeding are frequently associated with higher DMFT scores [1,2]. No study has examined dental history in conjunction with sociodemographic data, DMFT, and BMI, amongst Saudi female students with high BMI. This investigation could provide us with a deeper understanding of the factors influencing oral health and identify potential areas for intervention.

The prevalence of dental caries, as measured by the DMFT index, is influenced by a variety of factors, including sociodemographic characteristics, such as age and education level [1,9]. Previous research demonstrated that sociodemographic factors significantly impact oral health outcomes [1,2,12,13]. For instance, individuals from lower socioeconomic backgrounds often exhibit higher DMFT scores, due to limited access to dental care, and lower health literacy [12,13]. Additionally, a high BMI has been associated with an increased risk of dental caries, potentially due to dietary habits and metabolic factors [13].

The relationship between BMI and oral health is complex and multifaceted. High BMI is often linked to poor dietary habits, including high sugar intake, which is a known risk factor for dental caries [1,2,14,15,16]. Moreover, metabolic changes associated with obesity can affect saliva composition and flow, further increasing the risk of caries [17]. The relationship between oral health and obesity amongst the Saudi population has been scarcely explored within the existing literature. One study observed no significant variation in BMI between adult patients with high and low dental caries at Taif University [18]. Conversely, another study identified a notable correlation between untreated dental caries and elevated BMI in children from Jazan [19]. Furthermore, it was reported that there was an inverse relationship between dental caries and BMI, noting that obese children exhibited better oral health [14]. Recently, in addition to the mean DMFT, scores were higher in obese children. This study suggests that salivary biomarker levels could be used as a non-invasive indicator to assess the obesity status of children [2]. Obesity is associated with metabolic changes, dietary patterns, and systemic inflammation, all of which may influence oral health [20]. Given the rising rates of obesity in Saudi Arabia, investigating the potential relationship between BMI and oral health outcomes is important. Previous studies have shown inconsistent findings regarding this association [1,2,9].

There is little available evidence on the assessment of high BMI patients’ oral health in the Saudi population, particularly among university female students. The sports center at Taif University provides a unique setting for this study, as it attracts a diverse group of female participants who engage in various physical activities. Understanding the relationship between their dental history, sociodemographic status, and DMFT index can provide valuable insights into the specific oral health challenges faced by this group. Therefore, to evaluate the association of high BMI (body mass index), dental history, diet, physical activity, and oral hygiene practices with DMFT (decayed, missing, and filled teeth) of female students at Taif University, Saudi Arabia.

## 2. Materials and Methods

### 2.1. Study Design, Location, and Time

A cross-sectional observational study was conducted from January to June 2023 at Taif University, Saudi Arabia. The study adhered to the Strengthening the Reporting of Observational Studies in Epidemiology (STROBE) guidelines.

### 2.2. Study Participants

The target population for this study comprised female students enrolled at Taif University during the 2022–2023 academic year. Participants were divided into two groups based on their BMI:

(1) **Control group** (BMI between 18.5 and 24.9 kg/m^2^) and
(2) **Obese group** (BMI ≥ 30 kg/m^2^), according to the World Health Organization (WHO) classification [21].

Among obese participants, further subgroups were created: Group I (BMI 30–39.9 kg/m^2^), Group II (BMI 40–44.9 kg/m^2^), and Group III (BMI ≥ 45 kg/m^2^). The inclusion criteria were female students aged 18–27 years, enrolled at the Taif University sports centre or attending the general university campus, who agreed to participate.

The exclusion criteria were pregnancy, systemic conditions known to affect oral health (except type 2 diabetes and asthma, which were analyzed separately), recent antibiotic use (within three months), or BMI outside the specified ranges.

### 2.3. Sampling Methodology

A convenience sampling method was used to recruit participants for this study. Recruitment was conducted via posters, flyers, and social media announcements targeting female students with BMIs both within the normal range (control group) and with obesity (obese group). Participants were screened for eligibility based on self-reported height and weight, which were subsequently confirmed through direct measurements.

A previous power analysis concluded that a minimum of 84 participants was necessary to detect a weak correlation (r = 0.3) between BMI and continuous variables at a 95% confidence level with an α error of 0.05%. Ultimately, 138 participants were included (86 obese, 52 controls).

### 2.4. Ethical Considerations

Ethical approval was obtained from the Institutional Review Board of Taif University (ethical clearance number: 44-131) and followed the principles of the Declaration of Helsinki. Written informed consent was obtained from all participants.

### 2.5. Data Collection

The data collection process consisted of two parts: an interview, followed by a clinical oral examination. The interview was conducted by examiners. The interview questions were divided into three sections. The first section collected sociodemographic information. The second section comprised ten closed-ended questions to gather information about current diet and physical activity. The third section included nine closed-ended questions to assess the participants’ oral hygiene practices and dental history. The interview comprehensively covered oral health impacts, medical history, oral symptoms, behavioral factors, and dietary habits. It detailed participants’ sociodemographic characteristics, such as age and parental education, and addressed oral hygiene practices, including brushing frequency and the use of fluoride toothpaste. Additionally, it examined the frequency of sugary food and beverage consumption and reviewed medical history, including the presence of systemic diseases and smoking status. It is important to note that dietary habits and oral hygiene practices were self-reported by participants based on memory and thus may be subject to recall bias.

Upon completion of the interview, each participant’s height (cm) and weight (kg) were measured, and BMI was calculated using the National Heart, Lung, and Blood Institute’s online BMI calculator (www.nhlbi.nih.gov). All participants wore gym suits and were instructed to remove accessories and shoes. The predetermined weight of the gym suit was subtracted from the measured weight. The interview was adapted from the Oral Health Impact Profile, a validated instrument that measures the impact of oral health on quality of life [22].

Clinical oral examinations were performed by a trained and calibrated dentist at the dental clinic of the sports facility. Each participant underwent a thorough clinical examination of the oral cavity. Primary teeth, early white spot lesions, fissure sealants, and third molars were excluded. Teeth missing for reasons other than carious extraction, such as orthodontic reasons or trauma, were considered healthy, in accordance with WHO criteria. Participants were seated comfortably on a removable dental chair. All teeth were air-dried using an air–water syringe, and cotton rolls were placed in the lingual and buccal sulcus for isolation. Basic examination instruments were used for the visual examination of the DMFT status.

### 2.6. Data Calculation

Descriptive and inferential data analyses were performed in this study. Descriptive statistics were used to describe participants’ characteristics, their responses to the questionnaire, and clinical examination. Descriptive statistics included frequencies and percentages for categorical variables, in addition to means and standard deviations, or medians and interquartile ranges, for continuous variables, depending on the data distribution. Inferential statistics were used to test the hypotheses and answer the research questions. Inferential statistics included contingency table arrays and the χ^2^ statistic for examining the association between categorical variables. Kolmogorov–Smirnov’s test was used to check that BMI was normally distributed. Independent *t*-tests and one-way ANOVA were used to compare BMI according to factors of 2 or more than 2 levels. Pearson or Spearman’s correlation coefficients were used to estimate the linear or non-linear correlation of BMI with other quantitative or ordinal variables. Mann–Whitney’s test was used to compare the distributions of ordinal variables between the groups. Fisher’s exact test was used to assess the association between categorical variables and groups. Linear regression analyses were conducted to identify potential predictors of dental caries and other oral health issues, adjusted for potential confounders. The associations between predictors and outcomes were presented as beta and 95% confidence intervals (CIs). All significance testing was two-tailed with an α *p* value of 0.05, and data were then analyzed using Stata Statistical Software Version 15.1 (release 15; StatCorp LLC, College Station, TX, USA).

## 3. Results

### 3.1. Sample Characteristics

A total of 138 female students participated in the study, with a mean age of 21 ± 2.4 years, 86 of whom were obese female students. Table 1 analyzed a sample of individuals categorized into a control group and an obese group. The BMI categories, shown in Table 2, were distributed as follows: 27.91% of participants had a BMI of 30–34.9 kg/m^2^, 40.70% had a BMI of 35–39.9 kg/m^2^, and 31.40% had a BMI of ≥40 kg/m^2^.

### 3.2. BMI in Relation to Dental History

When analyzing data in Table 3, the reasons for visiting the dentist across different BMI groups yielded significant findings. Out of a total sample of 86 participants, the chief complaints were then analysed for 21 individuals. Participants with a BMI of 40–45 kg/m^2^ and those with a BMI greater than 45 kg/m^2^ were more likely to visit the dentist for examinations and pain compared to those with a BMI of less than or equal to 40 kg/m^2^. Specifically, 22.2% of participants in the 40–45 kg/m^2^ group, and 11.1% in the greater than 45 kg/m^2^ group visited for examinations, whereas none in the less than or equal to 40 kg/m^2^ group did (*p* < 0.05). Pain was the predominant reason for dental visits across all BMI groups, with 100% of participants in the less than or equal to 40 kg/m^2^ group, 77.8% in the 40–45 kg/m^2^ group, and 77.8% in the greater than 45 kg/m^2^ group, reporting pain as their main reason behind the dental visit (*p* < 0.05). Additionally, swelling and pain were reported by 11.1% of participants in the greater than 45 kg/m^2^ group, whilst no participants within the other BMI groups reported this reason (*p* < 0.05). These findings underscore the varying reasons for dental visits among different BMI groups, with pain being a common factor across all groups. Follow-up visits were exclusive to the control group (48.8%), suggesting a lack of post-treatment continuity among obese individuals. Swelling and pain were reported only in the ≥ 45 kg/m^2^ group (11.1%).

### 3.3. BMI in Relation to Medical, Behavioural and Social Characteristics

The results demonstrated (Table 4) that the incidence of systemic diseases was significant, with 11.6% of obese participants diagnosed with asthma, and an additional 11.6% diagnosed with type 2 diabetes. Additionally, it was observed that the prevalence of type 2 diabetes increased significantly within the highest BMI level group, reaching 33.3%.

These results also revealed that the smoking prevalence within the obese sample was 7.0% compared with 1.9% in the control group. As shown in Table 5, parental educational attainment was also recorded, indicating that 34.1% of fathers and 39.9% of mothers had completed university-level education. With our population being Saudi university students, this research is primarily exploratory. Other studies assessing BMI patients have provided similar sample sizes [23,24]. Furthermore, the vast majority of obese participants, 75.6%, originated from rural areas (Table 6).

Table 7 summarizes the relationship between BMI and various brushing behaviors amongst the participants. The majority of participants across all BMI categories used a toothbrush, with no significant difference observed between the groups (*p* = 0.606). Miswak usage was relatively consistent, and a small percentage of participants used both methods. Regarding brushing frequency, most participants brushed their teeth once daily, regardless of their BMI. The frequency of brushing twice daily was similar across all of the BMI categories, showing no significant correlation (rS = 0.02; *p* = 0.880). Fluoride toothpaste was predominantly used across all BMI groups, with no significant variation noted (*p* = 0.690). A high percentage of participants reported never visiting a dentist, with no significant difference observed between BMI groups (*p* = 0.366). Lastly, the daily frequency of healthy and processed food consumption showed no significant correlation with BMI (rS = −0.01; *p* = 0.958 for healthy food and rS = −0.08; *p* = 0.460 for processed food).

### 3.4. BMI in Relation to DMFT Index

The relationship between the DMFT (Decayed, Missing, and Filled Teeth) Index and BMI amongst the participants is summarized in Table 8. The mean number of decayed teeth was at its highest in participants with a BMI of ≤40 kg/m^2^ (5.79), and lowest in those with a BMI of 40–45 kg/m^2^ (5.31). However, the correlation between BMI and decayed teeth was not statistically significant (r = −0.03; *p* = 0.781).

For missing teeth, participants with a BMI > 45 kg/m^2^ had the highest mean number (1.85), while those with a BMI ≤ 40 kg/m^2^ had the lowest (1.25). Despite this variation, the correlation was not statistically significant (r = 0.20; *p* = 0.065).

The mean number of filled teeth was highest in participants with a BMI of 40–45 kg/m^2^ (0.94), and lowest in those with a BMI > 45 kg/m^2^ (0.44). This correlation was also not significant (r = −0.06; *p* = 0.593).

Overall, the DMFT index showed no significant correlation with increasing BMI (r = 0.03; *p* = 0.782), with mean values ranging from 7.50 to 8.06 across different BMI categories (Table 8).

### 3.5. Association Between DMFT and BMI Adjusted for Age and Diabetes Mellitus Type 2

**Figure 1:**
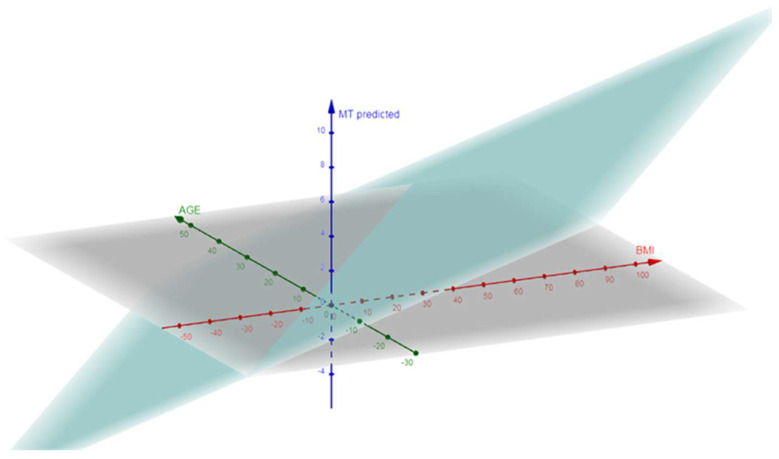
Three-dimensional scatter plot showing the relationship between age, BMI, and predicted missing teeth (MT). Note that the plane MT shows an increasing slope as the BMI increases and a negative slope as the age increases.

The multiple linear regression analysis revealed no significant association between the DMFT index and BMI (β = 0.06, 95% CI: −0.18 to 0.30, *p* = 0.616), age (β = 0.06, 95% CI: −0.34 to 0.46, *p* = 0.759), or diabetes mellitus type 2 (β = −1.47, 95% CI: −4.55 to 1.61, *p* = 0.346) (Table 9). However, a significant positive association was found between missing teeth (MT) and BMI (β = 0.09, 95% CI: 0.00 to 0.18, *p* = 0.045), indicating that a higher BMI is associated with an increase in the number of missing teeth. The association between missing teeth, and age suggested a significance (β = −0.14, 95% CI: −0.29 to 0.01, *p* = 0.056), while no significant association was observed with diabetes mellitus type 2 (β = 0.26, 95% CI: −0.88 to 1.40, *p* = 0.649) (Table 9) (Figure 1). A multiple linear regression analysis adjusting for age showed that BMI remained significantly associated with missing teeth (β = 0.09, 95% CI: 0.00–0.18, *p* = 0.045), while age demonstrated a borderline association (*p* = 0.056).

## 4. Discussion

The main aim of this study was to investigate the relationship between patients’ dental history, sociodemographic data, and DMFT index, amongst the female students at Taif University sports center with high BMI. The findings provide valuable insights into the complex interplay between obesity and oral health in this specific population. An important strength of this study is the inclusion of a control group of students with normal BMI, allowing direct comparison with obese participants. The comparative analysis revealed that obesity was associated with a higher prevalence of systemic diseases, more frequent pain-related dental visits, and an increased number of missing teeth.

### 4.1. Dental History and BMI

Our data analysis demonstrated significant differences in the reasons for dental visits across the different BMI groups. Participants with a higher BMI (40–45 kg/m^2^ and >45 kg/m^2^) were more likely to visit the dentist for examinations and pain, compared to those with a lower BMI of ≤40 kg/m^2^. These results align well with previous research, which indicates that individuals with a higher BMI are more prone to dental-related issues, possibly due to dietary habits and metabolic factors associated with obesity [25]. Pain was the predominant reason for dental visits across all BMI groups, highlighting the need for targeted interventions to address pain management in patients with defined obesity.

### 4.2. Medical, Behavioral, and Social Characteristics

This study also showed a significant incidence of systemic diseases amongst the participants, such as asthma and type 2 diabetes. The prevalence of type 2 diabetes was notably higher in the highest BMI group, consistent with the existing literature, which links obesity with an increased risk of chronic diseases [26,27]. Smoking prevalence was relatively low, and parental educational attainment varied, with a substantial proportion of participants originating from rural areas. These sociodemographic factors are crucial in understanding the broader context of health behaviors and access to healthcare services [1,9,14].

To our surprise, the study found no significant correlation between BMI and dental hygiene practices or dietary habits. A total of 60.5% of our participants brushed their teeth once daily with a fluoride toothpaste, regardless of their BMI. The aforementioned results are similar to those previously reported by Alghamdi et al. [2]. At the same time, 21% of our participants brushed their teeth twice a day. This is also similar to previous reported literature, which showed that 27.5% of participants brushed their teeth twice daily [9]. This contrasts with a study [28], which found that 62% of Kuwaiti adults brushed their teeth twice daily. Our results suggest that whilst BMI may influence the frequency and reasons for dental visits, it does not necessarily impact daily oral hygiene practices. The lack of significant correlation between BMI and dietary habits also indicates that other factors, such as metabolic changes and systemic health conditions, may play a more critical role in the relationship between obesity and oral health.

### 4.3. DMFT Index and BMI

The relationship between the DMFT index and BMI was not statistically significant, although participants with a BMI ≤ 40 kg/m^2^ had the highest mean number of decayed teeth. Our results correlate with previous findings, which reported that the BMI of adult patients at Taif University, with high or low incidence of dental caries, was not significantly different [18]. In contrast, two Saudi studies conducted in Jeddah and Jazan reported a positive correlation between oral health and BMI [1,19]. The significant positive association between missing teeth and higher BMI suggests that obesity may contribute to caries-related tooth loss or other oral health complications [29]. Participants with higher BMI were more likely to have elevated DMFT scores, even after accounting for other factors such as low education, edematous gingiva, smoking, and medical conditions [1]. Obesity is associated with systemic inflammation and metabolic changes that can affect oral health [30,31]. For instance, higher levels of body fat can lead to increased levels of inflammatory markers, which may contribute to oral health issues such as caries and periodontal disease [32]. In a study conducted at another Saudi university, Jouhar et al. (2021) [33] identified a significant correlation between the DMFT index and obesity among students. The prevalence of dental caries was highest among overweight (DT = 2.80 ± 2.39) and obese (DT = 4.50 ± 0.985) participants [33]. Additionally, the study found that the associations between BMI and decayed teeth, filled teeth, and the overall DMFT index remained highly significant after adjusting for confounders in the linear regression model [33]. Researchers [34] reported that 6-year-old children who were overweight or obese exhibited a 1.92-fold and 3.6-fold increased risk of developing dental caries, respectively. Additionally, the study found that 13-year-old children in the same cohort who were overweight or obese had a 1.68-fold and 1.8-fold higher risk of dental caries compared to their normal-weight peers. Younger individuals are more prone to developing dental decay due to their higher consumption of sweets and lower intake of natural dairy products.

The lack of significant association between the DMFT index and BMI after consideration of age and diabetes mellitus type further underscores the multifaceted nature of this relationship. Even after adjusting for age, higher BMI remained a significant predictor of missing teeth. This finding is consistent with some studies conducted among university populations, although other research has suggested that age might attenuate this association [29]. Type 2 diabetes is associated with higher blood glucose levels, which can lead to an increased risk of dental caries due to the presence of glucose in saliva, providing a substrate for cariogenic bacteria [35,36]. The discrepancy in the findings may be due to differences in oral health indicators, BMI categories, sample size, and confounding factors controlled for in the analyses. Further studies are required to clarify the relationship between oral health and BMI in various settings and age groups.

### 4.4. Implications for Public Health and Future Research

The findings of this study highlight the importance of considering BMI as a factor in oral health assessments and interventions. Given the rising prevalence of obesity in Saudi Arabia, particularly amongst women, targeted public health strategies are required to address the unique oral health challenges faced by this population [9,10]. Future research should focus on longitudinal studies to better understand the causal relationships between obesity and oral health outcomes. Additionally, exploring the role of dietary habits, metabolic factors, and access to dental care in this context could provide more comprehensive insights.

### 4.5. Study Limitations

Several limitations should be considered when interpreting the results of this study. Firstly, this study was limited to female participants due to the strict gender segregation and cultural sensitivities in the Kingdom of Saudi Arabia. As a result, the findings are not applicable to the male population. The cross-sectional study design and convenience sampling methods employed in this research position it at the lower end of the evidence hierarchy, thus preventing the establishment of causal relationships between the identified variables. Dental caries were diagnosed solely through visual examination, without the use of radiographic confirmation. Additionally, the self-reported nature of dietary and oral hygiene data introduces potential recall bias, which may affect the accuracy of the findings. Moreover, the single-center nature of the study introduces potential bias related to local demographics, healthcare practices, and institutional factors. As a cross-sectional study, it is challenging to establish causal relationships, and the observed associations may be influenced by other unexamined factors. Recall bias related to genetic and environmental causes cannot be excluded. Furthermore, as this was a pilot study, the sample size was relatively small, which may limit the statistical power to detect significant findings and could result in overestimated odds ratios (ORs). To enhance the current understanding, further research with a larger and more inclusive sample size of other high-risk populations is recommended.

## 5. Conclusions

In conclusion, this study highlights the complex relationship between obesity and oral health amongst female students at Taif University. By comparing obese participants to a normal BMI control group, we found that a higher BMI was associated with more frequent dental visits, mainly driven by pain, and a higher prevalence of systemic diseases such as asthma and type 2 diabetes. While no significant correlation was observed between BMI and daily oral hygiene practices or dietary habits, a positive association between higher BMI and missing teeth suggests that obesity may contribute to caries-related tooth loss. These findings underscore the importance of integrating BMI considerations into dental health assessments and public health strategies, particularly in regions with rising obesity rates. Future research should utilize larger, more diverse populations and explore potential metabolic and behavioral mechanisms linking obesity and oral health outcomes.

## Figures and Tables

**Table 1:** Details of the study sample.

Variables	Control Group	Obese Group
	Mean ± S.D.	Mean ± S.D.
**Age**	20.3 (±1.2)	21 (±2.4)
**Height**	1.59 (±0.05)	1.54 (±0.07)
**Weight**	52.2 (±5.8)	102 (±11.9)
**Body Mass Index (BMI)**	20.7 (±2.1)	42.8 (±4.3)
**Number of Teeth**	27.6 (±0.9)	26.6 (±1.5)
**Decayed Teeth (D)**	4.37 (±3.4)	5.5 (±3.9)
**Missing Teeth (M)**	0.62 (±1.2)	1.6 (±1.6)
**Filled Teeth (F)**	1.8 (±2.7)	0.7 (±1.3)
**DMFT score**	7.4 (±4.2)	7.8 (±4.2)

**Table 2:** Distribution of body mass index (BMI) categories among control and obese groups.

	Total		Control		Obese	
	N	%	N	%	N	%
**Total**	138	100.0	52	100.0	86	100.0
**≤40 kg/m^2^**	76	55.1	52	100.0	24	27.9
**40–45**	35	25.4	0	0	35	40.7
**>45 kg/m^2^**	27	19.6	0	0	27	31.4

**Table 3:** BMI in relation to dental history.

			BMI Range							
	Control		30–39.9 kg/m^2^		40–44.9 kg/m^2^		>45 kg/m^2^		Total	
	N	*%*	N	%	N	%	N	%	N	%
Total	41	*100.0*	3	100.0	9	100.0	9	100.0	62	100.0
Examination	4	*9.8*	0	0.0	2	22.2	1	11.1	7	11.3
Pain	17	*41.5*	3	100.0	7	77.8	7	77.8	34	54.8
Swelling and pain	0	*0.0*	0	0.0	0	0.0	1	11.1	1	1.6
Follow-up	20	*48.8*	0	0.0	0	0.0	0	0.0	20	32.3

**Table 4:** BMI in relation to systemic diseases.

			BMI Range							
	Control		30–39.9 kg/m^2^		40–44.9 kg/m^2^		>45 kg/m^2^		Total	
	N	%	N	%	N	%	N	%	N	%
Total	52	100.0	24	100.0	35	100.0	27	100.0	138	100.0
Healthy	48	92.3	19	79.2	30	85.7	17	63.0	114	82.6
Asthma	2	3.8	4	16.7	5	14.3	1	3.7	12	8.7
Diabetes type 2	1	1.9	1	4.2	0	0.0	9	33.3	11	8.0

**Table 5:** Relationship between parents’ education and BMI of the participants.

				BMI Range								*p* Value Results of the Spearman’s Rank Correlation Coefficient and F-Test ANOVA
		Control		30–39.9 kg/m^2^		40–44.9 kg/m^2^		>45 kg/m^2^		Total		
		N	%	N	%	N	%	N	%	N	%	
**Father Education**	**Total**	52	100.0	24	100.0	35	100.0	27	86	138	100.0	rS = −0.09; *p* = 0.413
	**None**	0	0	1	4.2	3	8.6	4	8	8	5.8	
	**Elementary**	5	9.6	7	29.2	6	17.1	6	19	24	17.4	
	**Primary**	4	7.7	3	12.5	5	14.3	6	14	18	13.0	
	**High school**	18	34.6	7	29.2	11	31.4	5	23	41	29.7	
	**University**	25	48.1	6	25.0	10	28.6	6	22	47	34.1	
**Mother Education**	**Total**	52	100.0	24	100.0	35	100.0	27	86	138	100.0	rS = 0.00; *p* = 0.981
	**None**	1	1.9	0	0.0	6	17.1	2	8	9	6.5	
	**Elementary**	6	11.5	4	16.7	7	20.0	7	18	24	17.4	
	**Primary**	6	11.5	6	25.0	4	11.4	6	16	22	15.9	
	**High school**	10	19.2	10	41.7	5	14.3	3	18	28	20.3	
	**University**	29	55.8	4	16.7	13	37.1	9	26	55	39.9	
**Parents Relationship**	**Total**	52	100.0	24	100.0	35	100.0	27	86	138	100.0	0.751 (F)
	**Married**	41	78.8	17	70.8	22	62.9	15	54	95	68.8	
	**Separated**	3	5.8	4	16.7	6	17.1	7	17	20	14.5	
	**F/M died**	8	15.4	3	12.5	7	20.0	5	15	23	16.7	

*p* < 0.05, BMI, body mass index, as a quantitative variable, was used in the analysis. BMI ranges were shown for descriptive purposes.

**Table 6:** The residency of the participants.

	Total		Control		Obese	
	N	%	N	%	N	%
Total	138	100.0	52	100.0	86	100.0
Rural	72	52.2	7	13.5	65	75.6
Village	22	15.9	1	1.9	21	24.4
City	44	31.9	44	84.6	0	0.0

**Table 7:** Association between body mass index (BMI) categories and brushing behavior based on two-sample *t*-test and Spearman’s rank correlation analysis.

				BMI Range								*p* Value Results of the 2-Sample *t*-Test and Spearman’s Rank Correlation Coefficient
		Control		30–39.9 kg/m^2^		40–44.9 kg/m^2^		>45 kg/m^2^		Total		
		N	%	N	%	N	%	N	%	N	%	
Brushing type	Total	52	100.0	19	100.0	31	100.0	22	100.0	124	100.0	0.606 (t)
	Brush	50	96.2	13	68.4	26	83.9	16	72.7	105	84.7	
	Miswak	2	3.8	5	26.3	5	16.1	6	27.3	18	14.5	
	Both	0	0.0	1	5.3	0	0.0	0	0.0	1	0.8	
Brushing frequency	Total	52	100.0	24	100.0	35	100.0	27	100.0	138	100.0	rS = 0.02; *p* = 0.880
	Never	1	1.9	6	25.0	4	11.4	6	22.2	17	12.3	
	Once	9	17.3	14	58.3	21	60.0	17	63.0	61	44.2	
	Twice	33	63.5	4	16.7	10	28.6	4	14.8	51	37.0	
Toothpaste Usage	Total	52	100.0	19	100.0	31	100.0	22	100.0	124	100.0	0.690 (t)
	Not using	2	3.8	7	36.8	9	29.0	8	36.4	26	21.0	
	Fluoride	46	88.5	12	63.2	22	71.0	14	63.6	94	75.8	
Frequency of dental visits	Total	52	100.0	24	100.0	35	100.0	27	100.0	138	100.0	0.366 (t)
	Never	8	15.4	21	87.5	26	74.3	18	66.7	73	52.9	
	Less than 3 times	28	53.8	3	12.5	4	11.4	7	25.9	42	30.4	
	I don’t know	3	5.8	0	0.0	5	14.3	2	7.4	10	7.2	
Daily frequency of healthy food	Total			24	100.0	35	100.0	27	100.0	86	100.0	rS = −0.01; *p* = 0.958
	Never			9	37.5	4	11.4	9	33.3	22	25.6	
	Once			8	33.3	19	54.3	12	44.4	39	45.3	
	Twice			4	16.7	6	17.1	3	11.1	13	15.1	
	Three times			3	12.5	6	17.1	3	11.1	12	14.0	
Daily frequency of processed food	Total			24	100.0	35	100.0	27	100.0	86	100.0	rS = −0.08; *p* = 0.460
	Once			3	12.5	5	14.3	4	14.8	12	14.0	
	Twice			5	20.8	5	14.3	10	37.0	20	23.3	
	Three times			12	50.0	16	45.7	9	33.3	37	43.0	

*p* < 0.05, BMI, body mass index, as a quantitative variable, was used in the analysis. BMI ranges were shown for descriptive purposes.

**Table 8:** Relationship between DMFT (decayed, missing, and filled teeth) index and BMI of the participants.

			BMI Range				*p* Value Results of Pearson’s r Correlation Coefficient
		Control	30–39.9 kg/m^2^	40–44.9 kg/m^2^	>45 kg/m^2^	Total	
Decayed Teeth (D)	Mean	4.37	5.79	5.31	5.44	5.07	r = −0.03; *p* = 0.781
	Standard Deviation	3.45	3.76	3.44	4.64	3.76	
Missing Teeth (M)	Mean	0.62	1.25	1.80	1.85	1.27	r = 0.20; *p* = 0.065
	Standard Deviation	1.24	1.03	1.83	1.77	1.57	
Filled Teeth (F)	Mean	1.81	0.46	0.94	0.44	1.09	r = −0.06; *p* = 0.593
	Standard Deviation	2.69	1.02	1.70	1.09	2.04	
DMFT	Mean	6.79	7.50	8.06	7.74	7.42	r = 0.03; *p* = 0.782
	Standard Deviation	4.25	4.04	4.13	4.66	4.26	

*p* < 0.05, BMI, body mass index, as a quantitative variable, was used in the analysis. BMI ranges were shown for descriptive purposes.

**Table 9:** Association between DMFT (decayed, missing, and filled teeth) index, missing teeth (MT), and BMI adjusted for age and diabetes mellitus type 2: findings from multiple linear regression.

	Beta	95% CI	*p*-Value (* *p* < 0.05)
**DMFT**			
BMI	0.06	−0.18 0.30	0.616
Age	0.06	−0.34 0.46	0.759
Diabetes Mellitus Type 2			
No	0		
Yes	−1.47	−4.55 1.61	0.346
**Missing Teeth (MT)**			
BMI	0.09	0.00 0.18	0.045 *
Age	−0.14	−0.29 0.01	0.056
Diabetes Mellitus Type 2			
No	0		
Yes	0.26	−0.88 1.40	0.649

* *p* < 0.05, BMI, body mass index, as a quantitative variable, was used in the analysis. BMI ranges were shown for descriptive purposes.

## Data Availability

The raw data supporting the conclusions of this article will be made available by the authors without undue reservation.
